# An Audacious Maneuver: First Record of *Leopardus guigna* in the Marine Environment

**DOI:** 10.3390/ani14192879

**Published:** 2024-10-06

**Authors:** Walter Sielfeld, Jonathan A. Guzmán, Arturo Clark, Juan Carlos Cubillos

**Affiliations:** 1 Taxa Gestión Ambiental en Recursos Naturales, Los Cóndores Y-27, Biobío 4451025, Chile; 2 Escuela de Educación, Departamento de Ciencias Básicas, Universidad de Concepción, Campus Los Ángeles, Juan Antonio Coloma Street, Nr. 2021, Los Ángeles 4451032, Chile; 3 Encargado de Coordinación Umag Tv Aysén y Vinculación, Centro Universitario Coyhaique, Universidad de Magallanes, Sede Regional Coyhaique, José Miguel Carrera Street, Nr. 485, Coyhaique 5951380, Chile; 4 Turismo Puerto Santo Domingo, Bahía Cubillos, Puerto Aisén 6000000, Chile

**Keywords:** Güiña, *Leopardus guigna*, swimming behavior, Patagonia, marine environments, conservation, seabirds, kelp forests

## Abstract

**Simple Summary:**

The Güiña (*Leopardus guigna*), the smallest Neotropical feline, is found in central and southern Chile and western Argentina. This communication documents the first known instance of a güiña swimming in a marine environment. The observation, made in the remote Refugio Channel in Northern Patagonia, Chile, suggests that this elusive species may utilize marine environments during their search for food, particularly during winter when terrestrial prey is scarce.

**Abstract:**

The Güiña (*Leopardus guigna*), the smallest Neotropical feline, inhabits central and southern Chile and western Argentina. This communication reports the first documented instance of a güiña swimming in a marine environment, observed in the Refugio Channel, which separates Refugio Island from the mainland in Northern Patagonia, Chile. In April 2023, a local resident recorded video footage of a güiña swimming near the eastern shore of the channel, emerging from the water, shaking off, and climbing a tree to groom itself. This observation suggests that the güiña might use the seacoast when searching for food, particularly during periods of low terrestrial prey availability during the winter. The ability of the güiña to adapt to such environments underscores the species’ ecological flexibility, previously undocumented in this context, and highlights the need for integrating marine resources into the species’ conservation strategies. The video’s quality is limited due to the simplicity of the recording device, but it provides crucial visual evidence of this behavior.

## 1. Introduction

The güiña (*Leopardus guigna*), also known as the kodkod, is the smallest member of the genus *Leopardus*, a group of small and medium-sized Neotropical felines within the “ocelot lineage” [1]. This species is distributed primarily across central and southern Chile from Los Vilos in the Coquimbo region to Laguna San Rafael in the Aysén region and extends to the westernmost areas of Argentina [2,3,4,5]. The güiña is known for its elusive nature and cryptic behavior, making it one of the least studied felines in the region. Morphologically, the güiña exhibits two main phenotypes: a common spotted form with yellowish fur and dark circular spots, and a rarer melanistic form characterized by entirely black fur [6,7,8].

Güiñas are typically associated with dense forest habitats, exhibiting nocturnal and crepuscular activity patterns [9,10]. Rivers and lakes often act as natural barriers influencing the movement of the species, gene flow, and hunting strategies [11]. Despite the species’ adaptability, knowledge about the güiña’s swimming capabilities, particularly in marine environments, is extremely limited. While there are documented cases of other felids, such as *Leopardus geoffroyi* and *Leopardus pardalis*, using freshwater habitats [12,13,14,15], güiñas had not previously been observed swimming in marine environments. This communication documents the first known instance of a güiña swimming in the Refugio Channel in Northern Patagonia, Chile, providing new insights into the species’ ecological plasticity and its potential to utilize marine resources.

The flexibility in habitat uses and ecological adaptation observed in *Leopardus guigna* is reminiscent of the adaptations seen in other elusive felid species, such as *Leopardus geoffroyi* and the snow leopard (*Panthera uncia*). Comprehensive works on the biology, behavior, and conservation status of the snow leopard have highlighted the species’ ability to survive in harsh and isolated environments, aided by breakthroughs in non-invasive genetics, camera traps, and GPS-satellite collaring [15,16,17]. In field studies of recent decades, camera traps have acquired particular significance for predators, including felines.

Like the snow leopard and *Leopardus geoffroyi*, the güiña’s ability to adapt to a variety of environments, including potentially utilizing marine resources, underscores the importance of advanced monitoring techniques and conservation strategies tailored to the species’ unique ecological requirements [9,16].

## 2. Materials and Methods

This observation was part of a broader study in the area surrounding the Refugio Channel (Punta Rasa: 43°57′22.3″ S–73°06′37.2″ W) located just north of Puerto Santo Domingo, in the Aysén Region of southern Chile, within boundaries of the Multiple Use Marine Protected Area known as (AMCP-MU Pitipalena—Añihue).

The rare sighting of the güiña swimming in the channel occurred on 7 April 2023, when a local resident, responsible for regularly checking the camera traps, was conducting one of his routine inspections. During this visit, he unexpectedly encountered the kodkod in the water and recorded the event using a basic mobile phone. Although the video footage is of limited quality due to the simplicity of the device (Video S1), it provides crucial visual evidence of this previously undocumented behavior. Future monitoring efforts will focus on improving the quality and coverage of the camera traps to better document such rare behaviors.

## 3. Results

**Figure 1:**
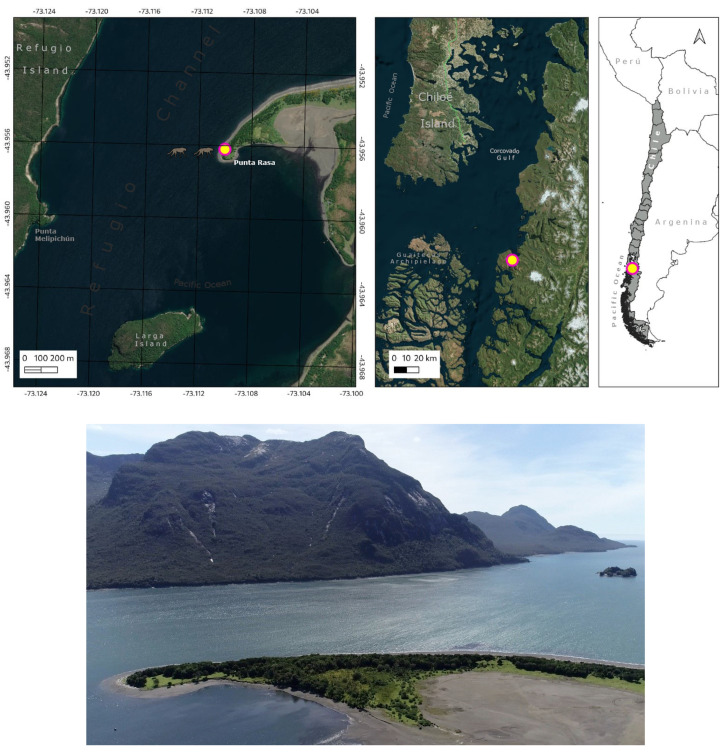
**Upper**: location of the study area and observation site in the Aysén Region, southern Chile. Yellow-pink dots mark the exact location of the record; the cat figure (brown) represents the güiña and its swimming direction. **Lower**: aerial view of the study area.

**Figure 2:**
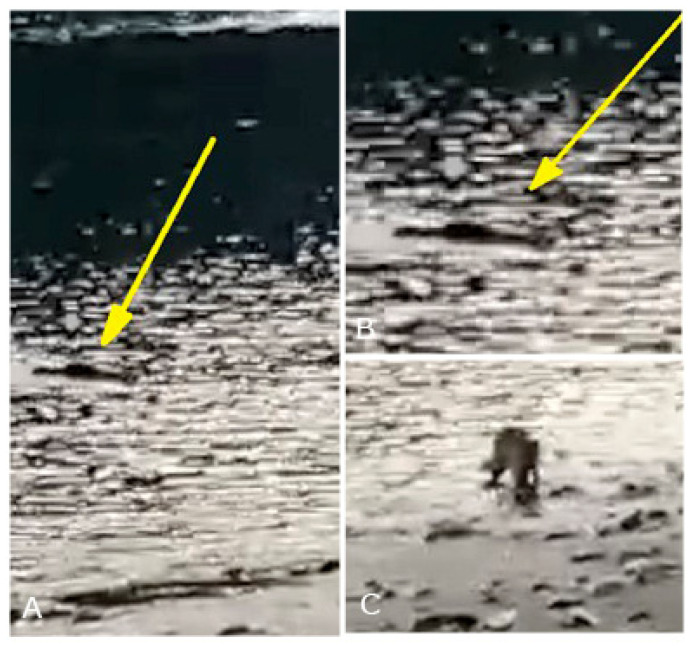
Sequence of still frames (screenshots from the video) showing the güiña swimming and emerging from the water. (**A**). Güiña swimming; (**B**). a close-up of photo A; (**C**). güiña emerging from the water. The video is available in the Supplementary Material (Video S1).

**Figure 3:**
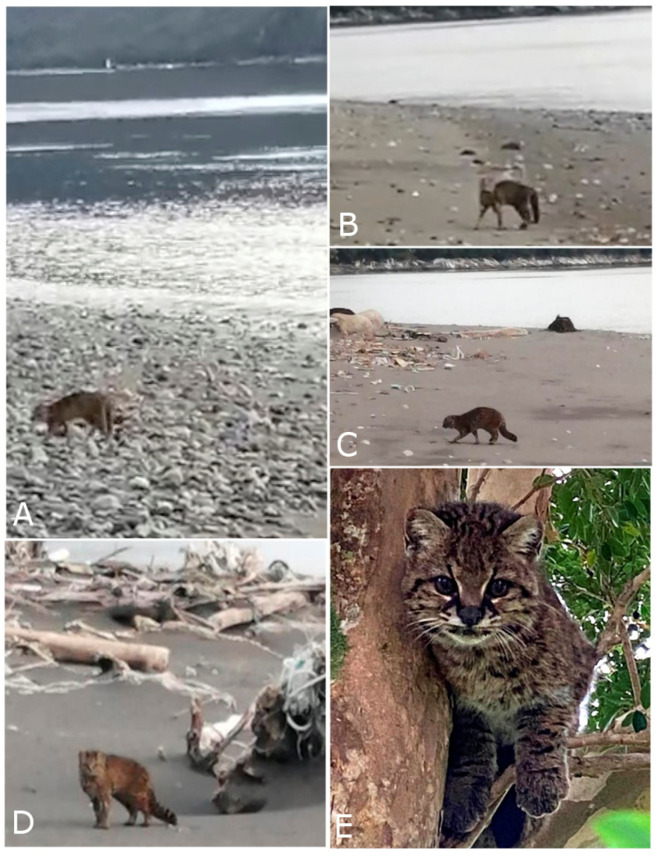
The güiña on the beach and later in a tree in the area. (**A**). In the first screenshot, the güiña is seen with Refugio Island in the background; (**B**–**D**). the güiña walking after emerging from the water; (**E**). the güiña in the tree. Screenshots taken from the video (Video S1).

This is the first documented case of a güiña in a marine environment. The observed güiña exhibited a mottled phenotype and appeared to be in good health, although its sex and age remain undetermined. The individual was observed swimming in the Refugio Channel (Figure 1) and then emerging on the eastern coast (Figure 2). This happened in the middle of the day from 2.00 pm to 3:00 pm during a casual and unscheduled observation. After exiting the water, the güiña shook itself, crossed a strip of beach of about 35 m from the tide line to the coastal forest (Figure 3A–D), and climbed a tree where it groomed itself (Figure 3E). The possibility of having accidentally fallen into the water was rejected given the gently sloping conditions in a large sector of the coastline surrounding the observation.

## 4. Discussion

This sighting marks the first known documentation of *Leopardus guigna* swimming in a marine environment, significantly broadening our understanding of the species’ ecological adaptability. The observation was made in an extremely remote and sparsely populated region of Patagonia, far from any urban centers (Figure 1). Given the species’ cryptic nature and the remoteness of the observation site, documenting such an unusual behavior is extraordinarily unlikely, which underscores the scientific importance of this finding.

Traditionally, the güiña has been associated with dense forest habitats, where its nocturnal and crepuscular behavior has made it particularly challenging to study [18,19,20,21]. However, this individual’s ability to swim in a marine environment suggests a possible adaptation to the unique conditions of the Northern Patagonian archipelago. Existing information about the güiña’s swimming behavior is limited, and previously documented observations have only referred to freshwater environments on the continent [12]. While the exact reasons for the güiña’s entry into the sea remain speculative, eventual swimming from the mainland and Refuge Island via the narrowest part of the channel (approximately 800 m, as shown in Figure 1) may be a possibility. This raises the question of its presence on other islands in the Northern Patagonian archipelago, particularly the Guaitecas Islands (44° S–74° W) [18,22]. Preliminary findings, including camera trap recordings from the surrounding continental region, indicate that the species regularly inhabits this area (Guzmán and Sielfeld in preparation).

The Refugio Channel (Figure 1), where this sighting occurred, hosts a rich diversity of marine life, including Imperial Cormorants (*Leucocarbo atriceps*) and Dominican Gulls (*Larus dominicanus*), which in the region’s feed on species associated to the kelp forests (*Macrocystis pyrifera*) [23]. It is possible that the güiña could prey at least occasionally on these and other littoral birds, particularly during winter when terrestrial prey may be less accessible due to snow cover. This behavior is consistent with the dietary flexibility observed in other terrestrial carnivores of Southern Patagonia, such as the Culpeo (*Lycalopex culpaeus*) and Chilla (*Lycalopex griseus*), which have been documented incorporating marine prey into their diets during periods of scarcity [12,24,25,26]. Although little is known about the predatory behavior of the güiña, this species, despite its small size, is considered a top predator, preying on small mammals, birds, and reptiles, which are all species significantly smaller than the cat itself [8,16,27,28,29,30,31]. Additionally, recent photographic evidence shows the species preying upon *Pudu puda* in southern Chile [32]. Thus, these new data not only expand our understanding of the güiña’s habitats but also its trophic niche.

Moreover, the ability of the güiña to swim aligns with behaviors observed in other felids in different contexts. For example, *Leopardus geoffroyi* and *Leopardus pardalis* have been observed swimming in rivers and lakes, using these environments as movement corridors or hunting grounds. Similarly, the Puma (*Puma concolor*) has been documented crossing rivers and lakes within its range, demonstrating significant swimming capabilities [10,11,12,15]. These parallels suggest that the güiña’s behavior may not be as anomalous as it first appears, indicating a broader pattern of aquatic adaptability among small felids.

While the video quality is limited due to the use of a basic mobile phone, the significance of this record lies in the rarity of the event and the direct evidence of previously undocumented behavior. Future research could benefit from more advanced recording equipment or enhanced camera traps to capture higher-quality footage, allowing for more detailed analysis of such behaviors. Continued monitoring of the region, especially using camera traps and other non-invasive methods, would be essential to gather more comprehensive data on the güiña’s behavior in marine environments and its potential use of biological corridors.

The potential crossing of the Refugio Channel by a güiña also raises intriguing questions about the species’ distribution and gene flow in the region. The possibility that güiñas might inhabit nearby islands, such as Refuge Island, has not been previously considered in phylogenetic studies [33]. If future studies confirm the presence of güiñas on these islands, it could indicate a previously unrecognized pattern of island colonization, suggesting that the species is more widely distributed in the Northern Patagonian archipelago than previously thought. This would also imply a higher degree of genetic flow between populations than currently assumed.

## 5. Conclusions

This communication provides the first evidence of a güiña in a marine environment. The eventual güiña’s ability to utilize marine resources, particularly during periods of low terrestrial prey availability, suggests a greater degree of ecological adaptability than previously recognized. Further research is needed to explore the extent of this behavior and its implications for the conservation of *Leopardus guigna*, particularly in the context of the Northern Patagonian archipelago.

This finding underscores the importance of including marine littoral environments in the conservation strategies for the güiña [20,21,34,35,36]. Given that the species is classified as “Near Threatened” by the Chilean Ministry of the Environment [37] and “Vulnerable” by the IUCN [19], it is crucial that conservation efforts consider not only fragmented or degraded terrestrial habitats but also the marine resources and corridors that could be vital for the species’ survival. The güiña’s adaptability to these environments suggests that its ecology is more complex and varied than previously understood, reinforcing the need for continued research and comprehensive conservation strategies that incorporate both terrestrial and marine ecosystems.

## Data Availability

The data presented in this study are available on request from the corresponding author. The video evidence of the kodkod swimming in the Refugio Channel is available as Supplementary Materials upon request.

## Supplementary Materials

The following supporting information can be downloaded at https://www.mdpi.com/article/10.3390/ani14192879/s1: Video S1: Güiña_Video.
